# The Inflammatory Milieu of Amniotic Fluid Increases with Chorio-Deciduitis Grade in Inflammation-Restricted to Choriodecidua, but Not Amnionitis, of Extra-Placental Membranes

**DOI:** 10.3390/jcm10143041

**Published:** 2021-07-08

**Authors:** Joon Hyung Lee, Chan-Wook Park, Kyung Chul Moon, Joong Shin Park, Jong Kwan Jun

**Affiliations:** 1 Department of Obstetrics and Gynecology, Seoul National University College of Medicine, Seoul 03080, Korea; kontractubex12@gmail.com (J.H.L.); jsparkmd@snu.ac.kr (J.S.P.); jhs0927@snu.ac.kr (J.K.J.); 2 Medical Research Center, Institute of Reproductive Medicine and Population, Seoul National University, Seoul 03080, Korea; 3 Department of Pathology, Seoul National University College of Medicine, Seoul 03080, Korea; blue7270@gmail.com

**Keywords:** chorio-deciduitis, grade, amnionitis, acute histologic chorioamnionitis, intra-amniotic inflammatory response

## Abstract

No information exists about whether intra-amniotic inflammatory response increases with a chorio-deciduitis grade in the context of both inflammation-restricted to chorio-decidua and amnionitis of extra-placental membranes among spontaneous preterm births. The objective of current study is to examine this issue. A study population included 195 singleton pregnant women with chorio-deciduitis, and who spontaneously delivered at preterm (21.6~35.7 weeks) within 7 days of amniocentesis. We examined intra-amniotic inflammatory response according to the chorio-deciduitis grade in the context of inflammation restricted to chorio-decidua and amnionitis of extra-placental membranes. Intra-amniotic inflammatory response was measured by MMP-8 concentration (ng/mL) and WBC-count (cells/mm^3^) in amniotic-fluid (AF). Inflammation restricted to chorio-decidua and amnionitis were present in 47.7% (93/195) and 52.3% (102/195) of cases, respectively. Median AF MMP-8 concentration and WBC-count significantly increased with chorio-deciduitis grade in the context of inflammation restricted to chorio-decidua. However, there was no significant difference in median AF MMP-8 concentration and WBC-count between chorio-deciduitis grade-1 and grade-2 in the context of amnionitis. The inflammatory milieu of AF increases with chorio-deciduitis grade in inflammation-restricted to chorio-decidua, but not amnionitis, of extra-placental membranes. This finding suggests that a chorio-deciduitis grade may have little effect on the intensification of intra-amniotic inflammatory response in the context of amnionitis of extra-placental membranes.

## 1. Introduction

Ascending intrauterine infection is a major pathophysiology of spontaneous preterm birth (PTB) [1,2,3,4,5,6,7,8,9,10,11,12,13]. It is well-known that intrauterine infection from the vaginal and cervical canals ascends to chorio-decidua (CD) and amnion in extra-placental membranes (EPM) [6,7,8,9], finally leading to fetal infection [1,2,3,4,5,6,7,8,9,10,11,12,13,14,15]. This traditional concept of ascending intrauterine infection suggests that the progression of intra-uterine infection is likely to cause the inflammatory responses of biological fluid (i.e., amniotic fluid (AF) and umbilical cord blood). Indeed, the previous studies demonstrated that intra-amniotic inflammatory response (IAIR) is significantly more intense in inflammation beyond CD (i.e., amnion or chorionic plate) than in inflammation restricted to CD [16,17,18,19]. Therefore, inflammation restricted to CD is known to be an early stage acute histologic chorioamnionitis (acute-HCA) while inflammation in the compartments beyond CD (i.e., amnion) is an advanced stage acute-HCA. This finding was reaffirmed by other previous studies as follows: (1) IAIR was more severe in patients with amnionitis than in those with only chorionitis [20,21,22,23]; (2) IAIR was more intense when inflammation was present in both chorionic plate and CD than when it was restricted to CD only, which was exposed to the cervical canal in placenta previa [24]. Moreover, IAIR increased according to the progression of inflammation in the detailed subdivisions of each placental compartment (i.e., EPM [25,26,27,28,29], umbilical cord [30], and chorionic plate [31]).

Although the intensity of IAIR increases with the total grade of acute-HCA [32], there is a paucity of information about which is more important between staging or grading in acute-HCA for the intensity of IAIR. In this regard, we previously demonstrated that advanced stage (i.e., inflammation in the compartments beyond CD) is associated with higher AF matrix metalloprotease-8 (MMP-8) concentrations and white blood cell (WBC) counts than early stage (i.e., inflammation restricted to CD) in the same context of acute-HCA total grade 2 [33]. However, no information exists about whether the inflammatory milieu of AF increases with chorio-deciduitis grade in the context of both inflammation restricted to CD and amnionitis of EPM. Based on the more importance of staging than grading, it is plausible that IAIR is not influenced by an increase of grade in chorio-deciduitis as a less advanced inflammation in the same context of amnionitis. The hypothesis of this study is that the inflammatory milieu of AF increases with chorio-deciduitis grade in inflammation restricted to CD, but not amnionitis, of EPM. The objective of the study is to examine this issue.

## 2. Materials and Methods

### 2.1. Study Design and Patient Population

This is a retrospective cohort study. Study population included 195 singleton pregnant women that met the following criteria: (1) delivered at Seoul National University Hospital between January 1993 and March 2007; (2) gestational age (GA) at delivery between 21.6 weeks and 35.7 weeks; (3) spontaneous PTB due to either preterm labor and intact membranes (PTL) or preterm premature rupture of membranes (preterm-PROM); (4) placental histology showing chorio-deciduitis; (5) no major fetal anomaly; and (6) delivered within 7 days of amniocentesis. This criterion of amniocentesis-to-delivery interval was used to preserve a meaningful temporal relationship between the results of AF and placental histopathologic findings. At our institution, amniocentesis for the retrieval of AF was routinely offered to all patients who were admitted with the diagnosis of either PTL or preterm-PROM for the identification of intra-amniotic infection or inflammation. Moreover, placental histologic examination was routinely offered and performed for all pregnant women who delivered at preterm due to either PTL or preterm-PROM. PTL and preterm-PROM were diagnosed with previously published criteria [34,35]. Written informed consent was gained from all study population. The Institutional Review Board of our institute specifically approved the current study (IRB number: 1909-120-106).

### 2.2. Clinical Characteristics and Pregnancy Outcomes

Clinical characteristics and pregnancy outcomes were obtained from a medical record review. Data included maternal age, parity, cause of preterm delivery, GA at amniocentesis and delivery, birth weight, gender of newborn, delivery mode, 1-min Apgar score, 5-min Apgar score, amniocentesis-to-delivery interval, antenatal use of antibiotics, gestational diabetes mellitus and suspected or proven early onset neonatal sepsis.

### 2.3. Diagnosis of Chorio-Deciduitis and Amnionitis

Placental tissue samples for pathologic examination included EPM (i.e., CD and amnion), chorionic plate and umbilical cord. These samples were fixed in 10% neutral buffered formalin and embedded in paraffin. Sections of prepared tissue blocks were stained with hematoxylin and eosin (H&E). Several pathologists were blinded to the clinical information related to placental tissues and examined the placental histopathology immediately after delivery. However, placental histo-pathologic examination was independently verified by a single pathologist (K.C.M.) who was also blinded to the clinical information between the year of 2017 and 2018. Grade 1 (mild) chorio-deciduitis was diagnosed in the presence of a least 1 focus of >5 neutrophils in the CD, and grade 2 (severe) chorio-deciduitis was diagnosed in the presence of diffuse neutrophilic infiltration in the CD; and amnionitis was diagnosed in the presence of at least 1 focus of >5 neutrophils in the amnion according to the criteria previously published [36].

### 2.4. The Studies of Amniotic Fluid (AF)

AF was cultured for aerobic and anaerobic bacteria, and genital mycoplasmas (*Ureaplasma urealyticum and Mycoplasma hominis*) and analyzed for WBC count according to the methods previously described [34,35]. The remaining fluid was centrifuged and stored in polypropylene tubes at −70 °C. MMP-8 concentrations in stored AF were measured with a commercially available enzyme-linked immunosorbent assay (Amersham Pharmacia Biotech, Inc., Little Chalfont, Bucks). The sensitivity of the test was <0.3 ng/mL. Both intra- and inter-assay coefficients of variation were <10%. Details about this assay and its performance were previously described [37]. IAIR was measured by MMP-8 concentration and WBC count in AF.

### 2.5. Early Onset Neonatal Sepsis

Early onset neonatal sepsis was diagnosed in the presence of a positive blood culture result within 3 days after birth. Early onset neonatal sepsis was suspected in the absence of a positive culture when two or more of the following criteria were present: (1) WBC count of <5000 cells/mm^3^; (2) polymorphonuclear leukocyte count of <1800 cells/mm^3^; and (3) I/T ratio (ratio of bands to total neutrophils) >0.2. These criteria have been previously used in the pediatric and obstetric literature [20]. Ten newborns were excluded from the assessment of early onset neonatal sepsis because they died immediately after birth due to extremely prematurity.

### 2.6. Statistical Analysis

**Figure 1:**
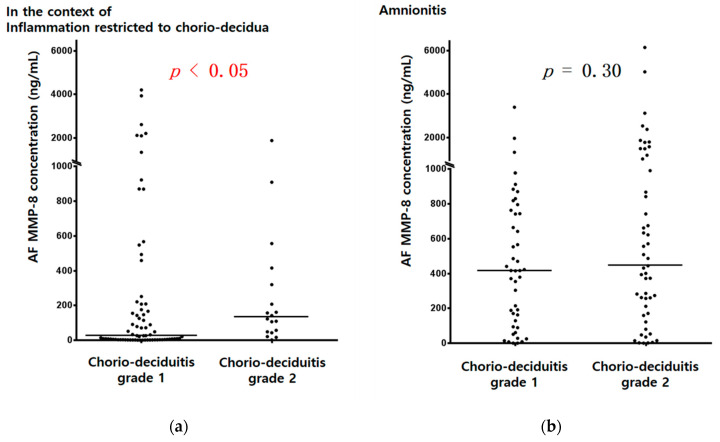
AF MMP-8 concentrations (ng/mL) according to chorio-deciduitis grade in the context of inflammation restricted to CD (**a**) (median, range; chorio-deciduitis grade 1: 26.0, (0.3, 4202.7); chorio-deciduitis grade 2: 131.9, (1.0, 1873.5); *p* < 0.05) and amnionitis (**b**) (median, range; chorio-deciduitis grade 1: 416.8, (0.3, 3392.0); chorio-deciduitis grade 2: 441.1, (0.4, 6142.6); Mann–Whitney U test, *p* = 0.30). Of 195 cases which met the entry for this study, 186 patients had an AF MMP-8 concentration; however, 9 patients did not have an AF MMP-8 concentration because of the limited amount of the remaining AF.

**Figure 2:**
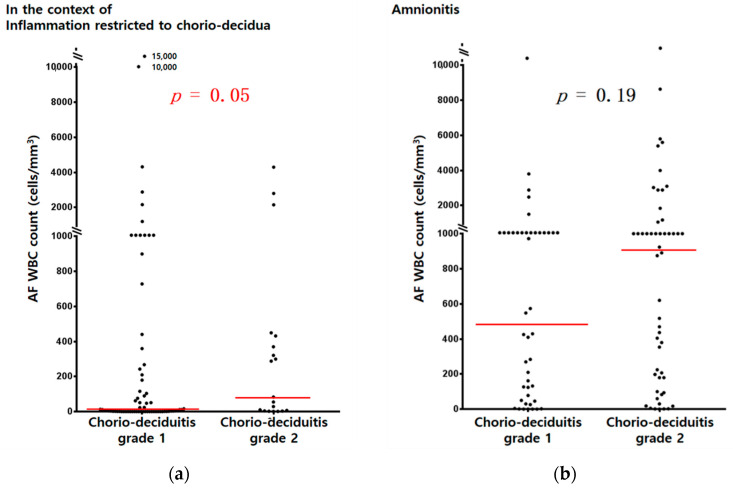
AF WBC counts (cells/mm^3^) according to chorio-deciduitis grade in the context of inflammation restricted to CD (**a**) (median, range; chorio-deciduitis grade 1: 9, (0, 15,000); chorio-deciduitis grade 2: 83 (1, 4300); *p* = 0.05) and amnionitis (**b**) (median, range; chorio-deciduitis grade 1: 490, (0, 13,428); chorio-deciduitis grade 2: 909 (0, 19,764); Mann–Whitney U test, *p* = 0.19). Of 195 cases which met the entry for this study, 185 patients had an AF WBC count; however, 10 patients did not have an AF WBC count because of the limited amount of AF.

**Figure 3:**
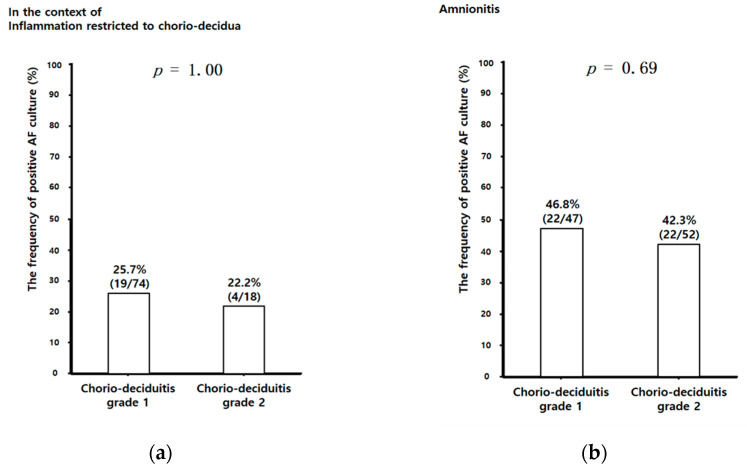
The frequency of positive AF culture according to chorio-deciduitis grade in the context of inflammation restricted to CD (**a**) (chorio-deciduitis grade 1: 25.7% (19/74); chorio-deciduitis grade 2: 22.2% (4/18); *p* = 1.00) and amnionitis (**b**) (chorio-deciduitis grade 1: 46.8% (22/47); chorio-deciduitis grade 2: 42.3% (22/52); Fisher’s exact test, *p* = 0.69). Of 195 cases which met the entry for this study, 191 patients had an AF culture result; however, 4 patients did not have an AF culture result because of the limited amount of AF.

Mann–Whitney U test was used for the comparison of continuous variables (Table 1 and Table 2, Figure 1 and Figure 2). Comparisons of proportions were performed with the Fisher’s exact test (Table 1 and Table 2, Figure 3). Statistical significance was defined as a *p* < 0.05.

## 3. Results

### 3.1. Clinical Characteristics and Pregnancy Outcomes According to Chorio-Deciduitis Grade in the Context of Inflammation Restricted to Chorio-Decidua (CD) and Amnionitis

Inflammation restricted to CD and amnionitis were present in 47.7% (93/195) and 52.3% (102/195) of study population, respectively, (Table 1 and Table 2). Table 1 and Table 2 demonstrated there was no significant difference in clinical characteristics and pregnancy outcomes between chorio-deciduitis grade 1 and grade 2 in the context of inflammation restricted to CD (Table 1) and amnionitis (Table 2).

### 3.2. Amniotic Fluid (AF) MMP-8 Concentrations and AF WBC Counts According to Chorio-Deciduitis Grade in the Context of Inflammation Restricted to Chorio-Decidua (CD) and Amnionitis

AF MMP-8 concentrations (ng/mL) (Figure 1a) and AF WBC counts (cells/mm^3^) (Figure 2a) were significantly higher in cases with chorio-deciduitis grade 2 than in those with chorio-deciduitis grade 1 in the context of inflammation restricted to CD. However, there was no significant increase in AF MMP-8 concentrations (Figure 1b) and AF WBC counts (cells/mm^3^) (Figure 2b) when chorio-deciduitis progressed from grade 1 to grade 2 in the context of amnionitis.

### 3.3. Early Onset Neonatal Sepsis According to Chorio-Deciduitis Grade in the Context of Inflammation Restricted to Chorio-Decidua (CD) and Amnionitis

In the context of inflammation restricted to CD, proven early onset neonatal sepsis was more frequent in cases with chorio-deciduitis grade 2 than in those with chorio-deciduitis grade 1 without reaching statistical significance (Table 1, 10.5% vs. 2.9%; *p* = 0.199). However, there was no significant difference in the frequency of proven early onset neonatal sepsis between chorio-deciduitis grade 1 and 2 in the context of amnionitis (Table 2, 6.2% vs. 6.2%; *p* = 1.000). These patterns correspond to those of IAIR (Figure 1 and Figure 2).

### 3.4. Positive Amniotic Fluid (AF) Culture According to Chorio-Deciduitis Grade in the Context of Inflammation Restricted to Chorio-Decidua (CD) and Amnionitis

Unlike AF MMP-8 concentrations and AF WBC counts, there was no significant difference in the frequency of positive AF culture between chorio-deciduitis grade 1 and grade 2 in the context of both inflammation restricted to CD (Figure 3a) and amnionitis (Figure 3b). We did not find the relationship between the type of specific organisms and chorio-deciduitis grade in the context of either inflammation restricted to CD or amnionitis. However, we consistently found genital mycoplasmas in more than 50% of positive AF culture in each group (data is not shown).

### 3.5. Histopathology According to Chorio-Deciduitis Grade in the Context of Inflammation Restricted to Chorio-Decidua (CD) and Amnionitis

**Figure 4:**
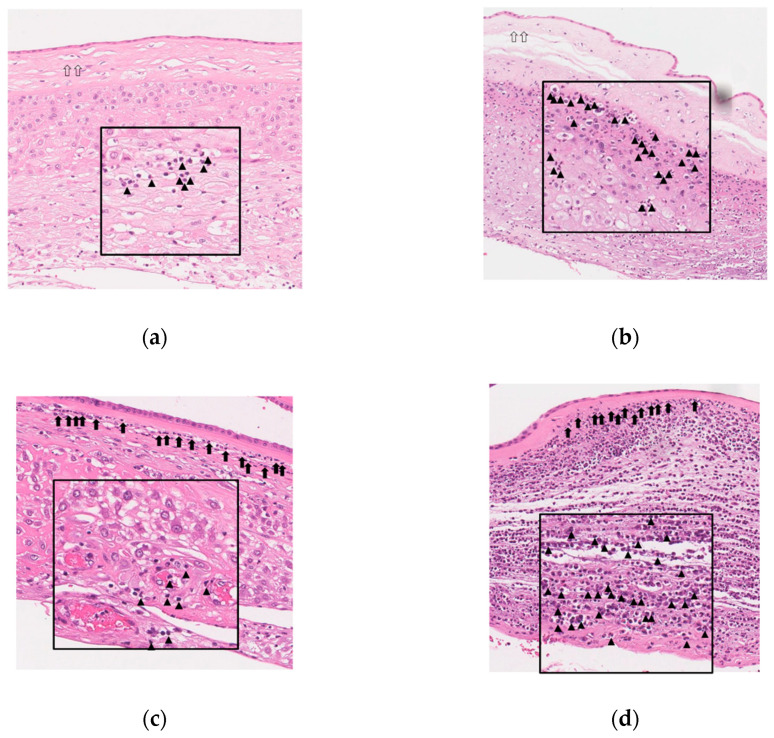
Histopathology according to chorio-deciduitis grade in the context of inflammation restricted to chorio-decidua (CD) and amnionitis. Hematoxylin and eosin-stained histologic sections of extra-placental membrane (EPM) are shown as follows: (**a**) chorio-deciduitis grade 1, inflammation restricted to CD; (**b**) chorio-deciduitis grade 2, inflammation restricted to CD; (**c**) chorio-deciduitis grade 1, amnionitis; and (**d**) chorio-deciduitis grade 2, amnionitis. These images are based on the magnification setting ×200, and the insets of panels are based on the magnification setting ×400. Open arrows indicate inflammation-free amnion (**a**,**b**), and black arrows show amnionitis with infiltrated neutrophils into amnion (**c**,**d**). Arrow heads indicate neutrophils infiltration into chorio-decidua (**a**–**d**).

Figure 4 shows representative images for chorio-deciduitis grade 1 in inflammation restricted to CD (Figure 4a), chorio-deciduitis grade 2 in inflammation restricted to CD (Figure 4b), chorio-deciduitis grade 1 in amnionitis (Figure 4c), and chorio-deciduitis grade 2 in amnionitis (Figure 4d) in H&E-stained histologic sections of EPM.

## 4. Discussion

**Figure 5:**
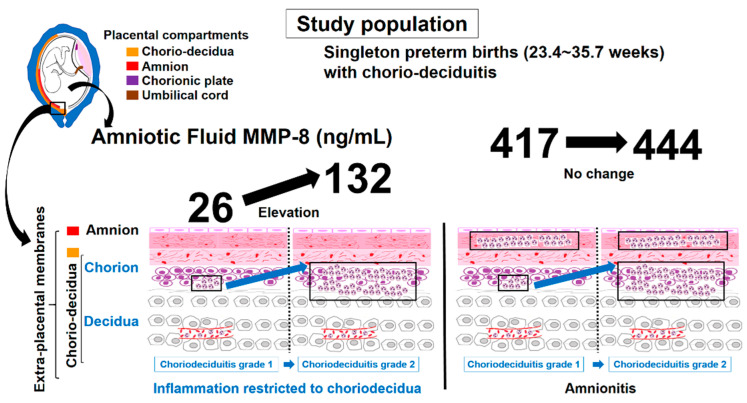
Schema of AF MMP-8 concentrations according to chorio-deciduitis grade in the context of inflammation restricted to chorio-decidua (CD) and amnionitis.

Principal finding of this study is that the inflammatory milieu of AF increases with chorio-deciduitis grade in inflammation restricted to CD, but not amnionitis, of EPM. This finding suggests that chorio-deciduitis grade may have little effect on the intensification of IAIR in the context of advanced stage acute-HCA (i.e., amnionitis) (Figure 5). This finding supports our previous assertion that the advanced compartment in the involved anatomical regions is more important than the grade by infiltrated neutrophils for the severity of IAIR in the progression of acute-HCA [33].

Our previous studies demonstrated IAIR increased according to the progression of inflammation in the detailed subdivisions of each placental compartment [25,26,27,28,29,30,31]. However, there is a paucity of data about the relationship between chorio-deciduitis grade and IAIR in the context of early stage and advanced stage acute-HCA in EPM. Moreover, very few previous studies about this issue had a limitation as in the following: (1) although only one previous study analyzed the relationship between positive AF culture and chorio-deciduitis grade in chorio-deciduitis, they did not control the presence of inflammation in other placental compartments (i.e., amnion) failing to exclude a major source of bias leading to a more inclusion of amnionitis in cases of higher chorio-deciduitis grade [38]; and (2) although another previous study examined the relationship between the total grade of acute-HCA and IAIR [32], that study did not examine on the effect of chorio-deciduitis grade on the intensity of IAIR. Indeed, we could not find any study controlling or adjusting for the stage (i.e., the advanced compartment in the involved anatomical regions of acute-HCA) in the analysis about the relationship between chorio-deciduitis grade and IAIR.

The conventional idea of ascending intrauterine infection depicts a model in which the micro-organism of cervical canal enters the decidua, followed by a widespread invasion of the chorion and amnion before crossing the intact membranes into the amniotic cavity [1,2,3,4,5,6,7,8,9,39,40,41,42,43]. However, one study using fluorescent in situ hybridization with a bacterial 16S rRNA probe demonstrated that focal infection of the CD in the vicinity of the cervical canal leads to intra-amniotic infection before the invasion of amnion and a diffuse inflammation of CD in the context of intact membranes [44,45]. Both mechanisms are plausible but there is insufficient evidence to determine which mechanism represents in vivo pathology of ascending intrauterine infection in humans. Nevertheless, it is well-known that intra-amniotic micro-organisms incite an IAIR resulting in an increase of chemokine level (e.g., CXCL6, IL-8) and chemotactic gradient [3,7,14,15]. Ultimately, this phenomenon causes amniotrophic outside-in neutrophil migration within EPM [7,14]. The extent of migration by neutrophils within EPM is thought to be dependent on the chemotactic gradient developed by chemokines concentration within AF, given that there is a stepwise increase in IAIR according to outside-in neutrophil migration from the decidua via the chorion to the amnion [20,21,22,23,25,26,27,28]. However, we should explain why the advanced compartment in involved anatomical regions (i.e., amnionitis) is more important than the chorio-deciduitis grade in EPM for the intensity of IAIR, and chorio-deciduitis grade may have little effect on the intensification of IAIR in the context of advanced stage acute-HCA (i.e., amnionitis). Our explanation is as follows (Figure 4). Firstly, neutrophils are likely to begin to gather in the CD (i.e., chorio-deciduitis grade 1 [focal aggregation] in the context of inflammation restricted to CD) in response to initial IAIR, and subsequently accumulate (i.e., chorio-deciduitis grade 2 [diffuse infiltration] in the context of inflammation restricted to CD) but still remain within CD according to a mild and significant increase of IAIR. Secondly, when IAIR surpasses a certain threshold, there is good chance that neutrophils within the CD migrate to the amnion leading to a subsequent decrease in the number of neutrophils in the CD, which means the regression from chorio-deciduitis grade 2 to chorio-deciduitis grade 1 (i.e., chorio-deciduitis grade 1 in the context of amnionitis). Finally, as only a secondary result of IAIR leading to amnionitis, neutrophils in the CD may be replenished from maternal decidual vessels resulting in an increase of chorio-deciduitis grade (i.e., chorio-deciduitis grade 2 in the context of amnionitis). However, all these explanations are only speculation because there is no clear animal or experimental model for the explanation of our current study’s results up to now. Therefore, further studies are needed for the elucidation of these issues.

In current study, the frequency of positive AF culture remained unaltered according to chorio-deciduitis grade in the context of both early and advanced stage acute-HCA. Although AF culture is the gold standard for the diagnosis of intra-amniotic infection, it is not a reliable proxy for IAIR as follows: (1) the frequency of positive AF culture remained low in clinical situation at high risk for ascending intrauterine infection such as PTL (10–13%) [46,47,48,49] and preterm-PROM (23–32%) [46,47,50,51,52]; (2) the footprint of micro-organism was identifiable even in the negative AF culture samples via molecular microbiologic techniques [53,54,55,56,57,58,59,60,61] implying the low sensitivity of culture technique; and (3) intra-amniotic inflammation, but not intra-amniotic infection, may be accompanied by an extra-amniotic infection in the early stage of ascending intrauterine infection, where micro-organisms reside in the CD. Therefore, AF culture results are unlikely to preserve the integrity of the inflammatory milieu of AF (i.e., AF MMP-8 and AF WBC count).

Major strengths of this study are as follows. Firstly, we controlled the involved placental compartments of acute-HCA (i.e., inflammation restricted to chorio-decidua, and amnionitis) for the analysis of the effect of chorio-deciduitis grade on the intensity of IAIR. This allowed us to assess the pure effect of chorio-deciduitis grade on the intensity of IAIR in the context of both early and advanced stage acute-HCA in EPM. Secondly, the intensity of IAIR was gauged with both AF MMP-8 concentration [62,63,64,65,66,67] and AF WBC count [34,35,68,69,70,71], well-known laboratory markers for IAIR in spontaneous PTB. These two markers showed consistent results adding to the credibility in current study. The limitations of this study are as follows. Firstly, this study is retrospective and has a small sample size. Secondly, chorio-deciduitis was not divided into detailed sub-divisions such as inflammation restricted to decidua, inflammation restricted to membranous trophoblast and inflammation in connective tissue of chorion. Thirdly, our study shows a huge variability in AF MMP-8 concentrations even in the same context of chorio-deciduitis grade 1 and grade 2 among patients with inflammation restricted to chorio-deciduitis or amnionitis. It is well-known that IAIR was greatly influenced by the grade and stage of placental inflammation. Moreover, GA at delivery [72] and the cause of PTB [73] have some influence on IAIR. Our previous studies demonstrated the relationship between GA at delivery and IAIR [72] and the relationship between the cause of PTB and IAIR [73] as in the following: (1) The inflammatory milieu of AF decrease in acute-chorioamnionitis with GA [72] and (2) IAIR is more severe in PTL than in preterm-PROM in the context of funisitis, despite less common positive AF culture [73]. Therefore, it is likely that AF MMP-8 concentrations are variable even in the same context of chorio-deciduitis grade 1 and grade 2 among patients with inflammation restricted to chorio-deciduitis or amnionitis, because GA at delivery is not the same and the cause of PTB is either PTL or preterm-PROM even in the same context of placental inflammatory condition. However, we did not adjust GA at delivery and the cause of PTB because GA at delivery and the cause of PTB were not significantly different between chorio-deciduitis grade 1 and grade 2 among patients with inflammation restricted to chorio-deciduitis or amnionitis (Table 1 and Table 2).

The classification of acute-HCA usually includes the stage (i.e., the location (compartment) of neutrophil infiltration) and grade (i.e., the degree of neutrophil infiltration in a specific compartment). However, we cannot find any studies examining the interaction between chorio-deciduitis grade and the advanced compartment (i.e., amnionitis) in the involved compartments of acute-HCA for the intensity of IAIR. To our knowledge, this is the first human study reporting that the severity of IAIR is higher in chorio-deciduitis grade 2 than chorio-deciduitis grade 1 in the context of early stage acute-HCA (i.e., inflammation restricted to CD), whereas in advanced stage acute-HCA (i.e., amnionitis), chorio-deciduitis grade 2 is not associated with a more severe IAIR than chorio-deciduitis grade 1. This finding may provide the obstetricians and researchers the information that chorio-deciduitis grade should not be overlooked in the context of early stage acute-HCA (i.e., inflammation restricted to CD) and may have little effect on the intensification of IAIR in the context of advanced stage acute-HCA (i.e., amnionitis).

The CD in itself is a large territory with the detailed sub-divisions composing of the outermost decidua, the membranous trophoblast of chorion as a middle layer, and the innermost connective tissue of chorion [25,26,27,28]. Our recent study has suggested that intra-amniotic inflammation is more frequent and intense according to outside-in neutrophil migration in the detailed subdivisions (i.e., the outermost decidua, the membranous trophoblast of chorion as a middle layer, and the innermost connective tissue of chorion) within the same CD [25]. Considering the results of our recent and current studies, neutrophils found in the innermost sub-divisional layer of CD (the connective tissue of chorion) is more likely to be associated with chorio-deciduitis grade 2 than chorio-deciduitis grade 1. Therefore, we should examine whether chorio-deciduitis grade 2 is associated with a more frequent neutrophil infiltration in the innermost connective tissue of chorion than chorio-deciduitis grade 1.

## 5. Conclusions

The inflammatory milieu of AF increases with chorio-deciduitis grade in early stage, but not advanced stage, acute-HCA in EPM. This finding suggests that chorio-deciduitis grade may have little effect on the intensification of IAIR in the context of advanced stage acute-HCA.

## Figures and Tables

**Table 1:** Clinical characteristics and pregnancy outcomes according to chorio-deciduitis grade in the context of inflammation restricted to chorio-decidua (CD).

	Chorio-Deciduitis Grade 1	Chorio-Deciduitis Grade 2	*p* ^†^
Inflammation restricted to CD (*n* = 93)	(*n* = 74)	(*n* = 19)	
Maternal age, years (mean ± SD)	30.0 ± 4.6	31.1 ± 3.3	0.230
Nulliparity	51.4% (38/74)	68.4% (13/19)	0.207
Causes of preterm birth			0.071
PTL	44.6% (33/74)	21.1% (4/19)	
Preterm-PROM	55.4% (41/74)	78.9% (15/19)	
GA at amniocentesis, (weeks) median, range	32.9 (23.0, 35.6)	32.6 (23.0, 35.6)	0.277
GA at delivery, (weeks) median, range	33.1 (23.4, 35.7)	32.7 (23.3, 35.7)	0.466
Amniocentesis-to-delivery interval, (hours) median, range	19.20 (0.01, 159.80)	53.30 (0.01, 152.80)	0.053
Birth weight, g (mean ± SD)	1854 ± 645	1691 ± 643	0.282
Male newborn	58.1% (43/74)	57.9% (11/19)	1.000
Cesarean section	33.8% (25/74)	36.8% (7/19)	0.793
Apgar score at 1 min <7	45.9% (34/74)	47.4% (9/19)	1.000
Apgar score at 5 min <7	28.4% (21/74)	15.8% (3/19)	0.381
Gestational diabetes mellitus	1.4% (1/74)	0% (0/19)	1.000
Antenatal use of antibiotics ^††^	60.3% (44/73)	84.2% (16/19)	0.061
Suspected early onset neonatal sepsis ^‡^	8.6% (6/70)	10.5% (2/19)	0.677
Proven early onset neonatal sepsis ^‡^	2.9% (2/70)	10.5% (2/19)	0.199
Suspected or proven early onset neonatal sepsis ^‡^	10.0% (7/70)	21.1% (4/19)	0.239

^†^ Mann–Whitney U test was used for the comparison of continuous variables and Fisher’s exact test was used for the comparison of proportions; ^††^ Of 93 cases, 92 patients were included in this analysis because the information about antenatal use of antibiotics in medical record was omitted in one patient; ‡ Four neonates were excluded from the analysis in the evaluation of early onset neonatal sepsis because they died shortly after delivery as a result of extremely prematurity and thus could not be evaluated with respect to the presence or absence of early onset neonatal sepsis; *NS*, not significant; *GA*, gestational age; *PTL*, preterm labor and intact membranes; *Preterm-PROM*, preterm premature rupture of membranes; *CD*, chorio-decidua.

**Table 2:** Clinical characteristics and pregnancy outcomes according to chorio-deciduitis grade in the context of amnionitis.

	Chorio-Deciduitis Grade 1	Chorio-Deciduitis Grade 2	*p* ^†^
Amnionitis (*n* = 102)	(*n* = 49)	(*n* = 53)	
Maternal age, years (mean ± SD)	30.4 ± 4.5	30.9 ± 4.6	0.573
Nulliparity	36.7% (18/49)	39.6% (21/53)	0.840
Causes of preterm birth			0.420
PTL	44.9% (22/49)	35.8% (19/53)	
Preterm-PROM	55.1% (27/49)	64.2% (34/53)	
GA at amniocentesis, (weeks) median, range	30.4 (24.1, 35.1)	29.1 (21.6, 35.1)	0.135
GA at delivery, (weeks) median, range	31.1 (24.1, 35.3)	29.3 (21.6, 35.7)	0.108
Amniocentesis-to-delivery interval, (h) median, range	32.80 (0.01, 163.70)	14.10 (0.01, 161.70)	0.503
Birth weight, g (mean ± SD)	1524 ± 501	1421 ± 584	0.260
Male newborn	46.9% (23/49)	39.6% (21/53)	0.549
Cesarean section	30.6% (15/49)	22.6% (12/53)	0.379
Apgar score at 1 min <7	61.2% (30/49)	64.2% (34/53)	0.839
Apgar score at 5 min <7	36.7% (18/49)	39.6% (21/53)	0.840
Gestational diabetes mellitus	2.0% (1/49)	5.7% (3/53)	0.619
Antenatal use of antibiotics ^††^	79.2% (38/48)	78.8% (41/52)	1.000
Suspected early onset neonatal sepsis ^‡^	25.0% (12/48)	20.8% (10/48)	0.809
Proven early onset neonatal sepsis ^‡^	6.2% (3/48)	6.2% (3/48)	1.000
Suspected or proven early onset neonatal sepsis ^‡^	31.2% (15/48)	27.1% (13/48)	0.823

^†^ Mann–Whitney U test was used for the comparison of continuous variables and Fisher’s exact test was used for the comparison of proportions; ^††^ Of 102 cases, 100 patients were included in this analysis because the information about antenatal use of antibiotics in medical record was omitted in two patients; ‡ Six neonates were excluded from the analysis in the evaluation of early onset neonatal sepsis because they died shortly after delivery as a result of extremely prematurity and thus could not be evaluated with respect to the presence or absence of early onset neonatal sepsis; *NS*, not significant; *GA*, gestational age; *PTL*, preterm labor and intact membranes; *Preterm-PROM*, preterm premature rupture of membranes; *CD*, chorio-decidua.

## Data Availability

Not applicable.

## References

[1] Goldenberg R.L., Hauth J.C., Andrews W.W. (2000). Intrauterine infection and preterm delivery. N. Engl. J. Med..

[2] Helmo F.R., Alves E.A.R., Moreira R.A.A., Severino V.O., Rocha L.P., Monteiro M.L.G.D.R., Reis M.A.D., Etchebehere R.M., Machado J.R., Corrêa R.R.M. (2018). Intrauterine infection, immune system and premature birth. J. Matern. Fetal Neonatal Med..

[3] Agrawal V., Hirsch E. (2012). Intrauterine infection and preterm labor. Semin. Fetal Neonatal Med..

[4] Kemp M.W. (2014). Preterm birth, intrauterine infection, and fetal inflammation. Front. Immunol..

[5] Chen H.J., Gur T.L. (2019). Intrauterine Microbiota: Missing, or the Missing Link?. Trends Neurosci..

[6] Romero R., Mazor M. (1988). Infection and preterm labor. Clin. Obstet. Gynecol..

[7] Cappelletti M., Presicce P., Kallapur S.G. (2020). Immunobiology of Acute Chorioamnionitis. Front. Immunol..

[8] Petit E., Abergel A., Dedet B. (2012). The role of infection in preterm birth. J. Gynecol. Obstet. Biol. Reprod..

[9] Menon R., Dunlop A.L., Kramer M.R., Fortunato S.J., Hogue C.J. (2011). An overview of racial disparities in preterm birth rates: Caused by infection or inflammatory response?. Acta Obstet. Gynecol. Scand..

[10] Gonçalves L.F., Chaiworapongsa T., Romero R. (2002). Intrauterine infection and prematurity. Ment. Retard. Dev. Disabil. Res. Rev..

[11] Stinson L.F., Payne M.S. (2019). Infection-mediated preterm birth: Bacterial origins and avenues for intervention. Aust. N. Z. J. Obstet. Gynaecol..

[12] Pavlidis I., Spiller O.B., Demarco G.S., MacPherson H., Howie S.E.M., Norman J.E., Stock S.J. (2020). Cervical epithelial damage promotes Ureaplasma parvum ascending infection, intrauterine inflammation and preterm birth induction in mice. Nat. Commun..

[13] Bayar E., Bennett P.R., Chan D., Sykes L., MacIntyre D.A. (2020). The pregnancy microbiome and preterm birth. Semin. Immunopathol..

[14] Kim C.J., Romero R., Chaemsaithong P., Chaiyasit N., Yoon B.H., Kim Y.M. (2015). Acute chorioamnionitis and funisitis: Definition, pathologic features, and clinical significance. Am. J. Obstet. Gynecol..

[15] Nadeau H.C., Subramaniam A., Andrews W.W. (2016). Infection and preterm birth. Semin. Fetal Neonatal Med..

[16] Park C.W., Kim S.M., Park J.S., Jun J.K., Yoon B.H. (2014). Fetal, amniotic and maternal inflammatory responses in early stage of ascending intrauterine infection, inflammation restricted to chorio-decidua, in preterm gestation. J. Matern. Fetal Neonatal Med..

[17] Abehsera D., Rodrigues Y., Mingorance J., Suárez A., Magdaleno F., Bartha J.L. (2014). Prediction and clinical relevance of pathologic patterns of injury associated with chorioamnionitis. Placenta.

[18] Buhimschi I.A., Zambrano E., Pettker C.M., Bahtiyar M.O., Paidas M., Rosenberg V.A., Thung S., Salafia C.M., Buhimschi C.S. (2008). Using proteomic analysis of the human amniotic fluid to identify histologic chorioamnionitis. Obstet. Gynecol..

[19] Hockney R., Waring G.J., Taylor G., Cummings S.P., Robson S.C., Orr C.H., Nelson A. (2020). Fetal membrane bacterial load is increased in histologically confirmed inflammatory chorioamnionitis: A retrospective cohort study. Placenta.

[20] Park C.W., Moon K.C., Park J.S., Jun J.K., Romero R., Yoon B.H. (2009). The involvement of human amnion in histologic chorioamnionitis is an indicator that a fetal and an intra-amniotic inflammatory response is more likely and severe: Clinical implications. Placenta.

[21] Yoneda S., Shiozaki A., Ito M., Yoneda N., Inada K., Yonezawa R., Kigawa M., Saito S. (2015). Accurate Prediction of the Stage of Histological Chorioamnionitis before Delivery by Amniotic Fluid IL-8 Level. Am. J. Reprod. Immunol..

[22] Kidokoro K., Furuhashi M., Kuno N., Ishikawa K. (2006). Amniotic fluid neutrophil elastase and lactate dehydrogenase: Association with histologic chorioamnionitis. Acta Obstet. Gynecol. Scand..

[23] Miura H., Ogawa M., Hirano H., Sanada H., Sato A., Obara M., Terada Y. (2011). Neutrophil elastase and interleukin-6 in amniotic fluid as indicators of chorioamnionitis and funisitis. Eur. J. Obstet. Gynecol. Reprod. Biol..

[24] Park C.W., Moon K.C., Park J.S., Jun J.K., Yoon B.H. (2009). The frequency and clinical significance of intra-uterine infection and inflammation in patients with placenta previa and preterm labor and intact membranes. Placenta.

[25] Oh J.W., Park C.W., Moon K.C., Park J.S., Jun J.K. Acute Chorioamnionitis and Intra-amniotic Inflammation are More Severe according to Outside-in Neutrophil Migration within the Same Chorio-decidua. Taiwan J. Obstet. Gynecol.

[26] Mauri A., Perrini M., Mateos J.M., Maake C., Ochsenbein-Koelble N., Zimmermann R., Ehrbar M., Mazza E. (2013). Second harmonic generation microscopy of fetal membranes under deformation: Normal and altered morphology. Placenta.

[27] Gupta A., Kedige S.D., Jain K. (2015). Amnion and Chorion Membranes: Potential Stem Cell Reservoir with Wide Applications in Periodontics. Int. J. Biomater..

[28] Avila C., Santorelli J., Mathai J., Ishkin S., Jabsky M., Willins J., Figueroa R., Kaplan C. (2014). Anatomy of the fetal membranes using optical coherence tomography: Part 1. Placenta.

[29] Park C.W., Oh J.W., Moon K.C., Park J.S., Jun J.K. (2017). Amniotic necrosis is associated with severe and advanced acute histologic chorioamnionitis. Placenta.

[30] Seong J.S., Park C.W., Moon K.C., Park J.S., Jun J.K. Necrotizing funisitis is an indicator that intra-amniotic inflammatory response is more severe and amnionitis is more frequent in the context of the extension of inflammation into Wharton’s jelly. Taiwan J. Obstet. Gynecol..

[31] Moon K.C., Oh J.W., Park C.W., Park J.S., Jun J.K. (2021). The Relationship Among Intra-Amniotic Inflammatory Response, The Progression of Inflammation in Chorionic Plate and Early-Onset Neonatal Sepsis. Front. Pediatr..

[32] Kim S.M., Romero R., Park J.W., Oh K.J., Jun J.K., Yoon B.H. (2015). The relationship between the intensity of intraamniotic inflammation and the presence and severity of acute histologic chorioamnionitis in preterm gestation. J. Matern. Fetal Neonatal Med..

[33] Park C.W., Yoon B.H., Kim S.M., Park J.S., Jun J.K. (2013). Which is more important for the intensity of intra-amniotic inflammation between total grade or involved anatomical region in preterm gestations with acute histologic chorioamnionitis?. Obstet. Gynecol. Sci..

[34] Yoon B.H., Jun J.K., Park K.H., Syn H.C., Gomez R., Romero R. (1996). Serum C-reactive protein, white blood cell count, and amniotic fluid white blood cell count in women with preterm premature rupture of membranes. Obstet. Gynecol..

[35] Yoon B.H., Yang S.H., Jun J.K., Park K.H., Kim C.J., Romero R. (1996). Maternal blood C-reactive protein, white blood cell count, and temperature in preterm labor: A comparison with amniotic fluid white blood cell count. Obstet. Gynecol..

[36] Yoon B.H., Romero R., Kim C.J., Jun J.K., Gomez R., Choi J.H., Syn H.C. (1995). Amniotic fluid interleukin-6: A sensitive test for antenatal diagnosis of acute inflammatory lesions of preterm placenta and prediction of perinatal morbidity. Am. J. Obstet. Gynecol..

[37] Park J.S., Romero R., Yoon B.H., Moon J.B., Oh S.Y., Han S.Y., Ko E.M. (2001). The relationship between amniotic fluid matrix metalloproteinase-8 and funisitis. Am. J. Obstet. Gynecol..

[38] Romero R., Salafia C.M., Athanassiadis A.P., Hanaoka S., Mazor M., Sepulveda W., Bracken M.B. (1992). The relationship between acute inflammatory lesions of the preterm placenta and amniotic fluid microbiology. Am. J. Obstet. Gynecol..

[39] Goldenberg R.L., Andrews W.W., Hauth J.C. (2002). Choriodecidual infection and preterm birth. Nutr. Rev..

[40] Grigsby P.L., Novy M.J., Waldorf K.M.A., Sadowsky D.W., Gravett M.G. (2010). Choriodecidual inflammation: A harbinger of the preterm labor syndrome. Reprod. Sci..

[41] Suff N., Karda R., Diaz J.A., Ng J., Baruteau J., Perocheau D., Tangney M., Taylor P.W., Peebles D., Buckley S.M.K. (2018). Ascending Vaginal Infection Using Bioluminescent Bacteria Evokes Intrauterine Inflammation, Preterm Birth, and Neonatal Brain Injury in Pregnant Mice. Am. J. Pathol..

[42] Waldorf K.M.A., Rubens C.E., Gravett M.G. (2011). Use of nonhuman primate models to investigate mechanisms of infection-associated preterm birth. BJOG Int. J. Obstet. Gynaecol..

[43] Fortner K.B., Grotegut C.A., Ransom C.E., Bentley R.C., Feng L., Lan L., Heine R.P., Seed P.C., Murtha A.P. (2014). Bacteria localization and chorion thinning among preterm premature rupture of membranes. PLoS ONE.

[44] Kim M.J., Romero R., Gervasi M.T., Kim J.S., Yoo W., Lee D.C., Mittal P., Erez O., Kusanovic J.P., Hassan S.S. (2009). Widespread microbial invasion of the chorioamniotic membranes is a consequence and not a cause of intra-amniotic infection. Lab. Investig..

[45] Jefferson K.K. (2012). The bacterial etiology of preterm birth. Adv. Appl. Microbiol..

[46] Romero R., Gómez R., Chaiworapongsa T., Conoscenti G., Kim J.C., Kim Y.M. (2001). The role of infection in preterm labour and delivery. Paediatr. Perinat. Epidemiol..

[47] Stranik J., Kacerovsky M., Andrys C., Soucek O., Bolehovska R., Holeckova M., Matulova J., Jacobsson B., Musilova I. (2021). Intra-amniotic infection and sterile intra-amniotic inflammation are associated with elevated concentrations of cervical fluid interleukin-6 in women with spontaneous preterm labor with intact membranes. J. Matern. Fetal Neonatal Med..

[48] Yoon B.H., Romero R., Moon J.B., Shim S.S., Kim M., Kim G., Jun J.K. (2001). Clinical significance of intra-amniotic inflammation in patients with preterm labor and intact membranes. Am. J. Obstet. Gynecol..

[49] Combs C.A., Gravett M., Garite T.J., Hickok D.E., Lapidus J., Porreco R., Rael J., Grove T., Morgan T.K., Clewell W. (2014). Amniotic fluid infection, inflammation, and colonization in preterm labor with intact membranes. Am. J. Obstet. Gynecol..

[50] Shim S.S., Romero R., Hong J.S., Park C.W., Jun J.K., Kim B.I., Yoon B.H. (2004). Clinical significance of intra-amniotic inflammation in patients with preterm premature rupture of membranes. Am. J. Obstet. Gynecol..

[51] Cobo T., Kacerovsky M., Palacio M., Hornychova H., Hougaard D.M., Skogstrand K., Jacobsson B. (2012). Intra-amniotic inflammatory response in subgroups of women with preterm prelabor rupture of the membranes. PLoS ONE.

[52] Cobo T., Kacerovsky M., Holst R.M., Hougaard D.M., Skogstrand K., Wennerholm U.B., Hagberg H., Jacobsson B. (2012). Intra-amniotic inflammation predicts microbial invasion of the amniotic cavity but not spontaneous preterm delivery in preterm prelabor membrane rupture. Acta Obstet. Gynecol. Scand..

[53] Han Y.W., Shen T., Chung P., Buhimschi I.A., Buhimschi C.S. (2009). Uncultivated bacteria as etiologic agents of intra-amniotic inflammation leading to preterm birth. J. Clin. Microbiol..

[54] Yoon B.H., Romero R., Kim M., Kim E.C., Kim T., Park J.S., Jun J.K. (2000). Clinical implications of detection of Ureaplasma urealyticum in the amniotic cavity with the polymerase chain reaction. Am. J. Obstet. Gynecol..

[55] Stinson L., Hallingström M., Barman M., Viklund F., Keelan J., Kacerovsky M., Payne M., Jacobsson B. (2020). Comparison of Bacterial DNA Profiles in Mid-Trimester Amniotic Fluid Samples from Preterm and Term Deliveries. Front. Microbiol..

[56] Keskin F., Ciftci S., Keceli S.A., Koksal M.O., Caliskan E., Cakiroglu Y., Agacfidan A. (2018). Comparison of culture and real-time polymerase chain reaction methods for detection of Mycoplasma hominis in amniotic fluids samples. Niger. J. Clin. Pract..

[57] Rodríguez N., Fernandez C., Zamora Y., Berdasquera D., Rivera J.A. (2011). Detection of Ureaplasma urealyticum and Ureaplasma parvum in amniotic fluid: Association with pregnancy outcomes. J. Matern. Fetal Neonatal Med..

[58] Yoon B.H., Romero R., Lim J.H., Shim S.S., Hong J.S., Shim J.Y., Jun J.K. (2003). The clinical significance of detecting Ureaplasma urealyticum by the polymerase chain reaction in the amniotic fluid of patients with preterm labor. Am. J. Obstet. Gynecol..

[59] Morimoto S., Usui H., Kobayashi T., Katou E., Goto S., Tanaka H., Shozu M. (2018). Bacterial-Culture-Negative Subclinical Intra-Amniotic Infection Can Be Detected by Bacterial 16S Ribosomal-DNA-Amplifying Polymerase Chain Reaction. Jpn. J. Infect. Dis..

[60] Marconi C., de Andrade Ramos B.R., Peraçoli J.C., Donders G.G., da Silva M.G. (2011). Amniotic fluid interleukin-1 beta and interleukin-6, but not interleukin-8 correlate with microbial invasion of the amniotic cavity in preterm labor. Am. J. Reprod. Immunol..

[61] DiGiulio D.B. (2012). Diversity of microbes in amniotic fluid. Semin. Fetal Neonatal Med..

[62] Park C.W., Yoon B.H., Kim S.M., Park J.S., Jun J.K. (2013). The frequency and clinical significance of intra-amniotic inflammation defined as an elevated amniotic fluid matrix metalloproteinase-8 in patients with preterm labor and low amniotic fluid white blood cell counts. Obstet. Gynecol. Sci..

[63] Revello R., Alcaide M.J., Dudzik D., Abehsera D., Bartha J.L. (2016). Differential amniotic fluid cytokine profile in women with chorioamnionitis with and without funisitis. J. Matern. Fetal Neonatal Med..

[64] Angus S.R., Segel S.Y., Hsu C.D., Locksmith G.J., Clark P., Sammel M.D., Macones G.A., Strauss J.F., Parry S. (2001). Amniotic fluid matrix metalloproteinase-8 indicates intra-amniotic infection. Am. J. Obstet. Gynecol..

[65] Kim A., Lee E.S., Shin J.C., Kim H.Y. (2013). Identification of biomarkers for preterm delivery in mid-trimester amniotic fluid. Placenta.

[66] Myntti T., Rahkonen L., Nupponen I., Pätäri-Sampo A., Tikkanen M., Sorsa T., Juhila J., Andersson S., Paavonen J., Stefanovic V. (2017). Amniotic Fluid Infection in Preterm Pregnancies with Intact Membranes. Dis. Markers.

[67] Myntti T., Rahkonen L., Pätäri-Sampo A., Tikkanen M., Sorsa T., Juhila J., Helve O., Andersson S., Paavonen J., Stefanovic V. (2016). Comparison of amniotic fluid matrix metalloproteinase-8 and cathelicidin in the diagnosis of intra-amniotic infection. J. Perinatol..

[68] Romero R., Quintero R., Nores J., Avila C., Mazor M., Hanaoka S., Hagay Z., Merchant L., Hobbins J.C. (1991). Amniotic fluid white blood cell count: A rapid and simple test to diagnose microbial invasion of the amniotic cavity and predict preterm delivery. Am. J. Obstet. Gynecol..

[69] Fan S.R., Liu P., Yan S.M., Peng J.Y., Liu X.P. (2020). Diagnosis and Management of Intraamniotic Infection. Matern. Fetal Med..

[70] Romero R., Yoon B.H., Mazor M., Gomez R., Diamond R.X., Kenney J.S., Ramirez M., Fidel P.L., Sorokin Y., Cotton D. (1993). The diagnostic and prognostic value of amniotic fluid white blood cell count, glucose, interleukin-6, and gram stain in patients with preterm labor and intact membranes. Am. J. Obstet. Gynecol..

[71] Abdel-Razeq S.S., Buhimschi I.A., Bahtiyar M.O., Rosenberg V.A., Dulay A.T., Han C.S., Werner E.F., Thung S., Buhimschi C.S. (2010). Interpretation of amniotic fluid white blood cell count in “bloody tap” amniocenteses in women with symptoms of preterm labor. Obstet. Gynecol..

[72] Park C.W., Park J.S., Jun J.K., Yoon B.H. (2015). The inflammatory milieu of amniotic fluid in acute-chorioamnionitis decreases with increasing gestational age. Placenta.

[73] Park C.W., Yoon B.H., Park J.S., Jun J.K. (2013). A fetal and an intra-amniotic inflammatory response is more severe in preterm labor than in preterm PROM in the context of funisitis: Unexpected observation in human gestations. PLoS ONE.

